# Structural and Biophysical Characterization of *Bacillus thuringiensis* Insecticidal Proteins Cry34Ab1 and Cry35Ab1

**DOI:** 10.1371/journal.pone.0112555

**Published:** 2014-11-12

**Authors:** Matthew S. Kelker, Colin Berry, Steven L. Evans, Reetal Pai, David G. McCaskill, Nick X. Wang, Joshua C. Russell, Matthew D. Baker, Cheng Yang, J. W. Pflugrath, Matthew Wade, Tim J. Wess, Kenneth E. Narva

**Affiliations:** 1 Dow AgroSciences, LLC, Indianapolis, Indiana, United States of America; 2 Cardiff School of Biosciences, Cardiff University, Cardiff, Wales, United Kingdom; 3 Rigaku Americas Corporation, The Woodlands, Texas, United States of America; 4 School of Optometry & Vision Sciences, Cardiff University, Cardiff, Wales, United Kingdom; University of Tennessee, United States of America

## Abstract

*Bacillus thuringiensis* strains are well known for the production of insecticidal proteins upon sporulation and these proteins are deposited in parasporal crystalline inclusions. The majority of these insect-specific toxins exhibit three domains in the mature toxin sequence. However, other Cry toxins are structurally and evolutionarily unrelated to this three-domain family and little is known of their three dimensional structures, limiting our understanding of their mechanisms of action and our ability to engineer the proteins to enhance their function. Among the non-three domain Cry toxins, the Cry34Ab1 and Cry35Ab1 proteins from *B. thuringiensis* strain PS149B1 are required to act together to produce toxicity to the western corn rootworm (WCR) *Diabrotica virgifera virgifera* Le Conte via a pore forming mechanism of action. Cry34Ab1 is a protein of ∼14 kDa with features of the aegerolysin family (Pfam06355) of proteins that have known membrane disrupting activity, while Cry35Ab1 is a ∼44 kDa member of the toxin_10 family (Pfam05431) that includes other insecticidal proteins such as the binary toxin BinA/BinB. The Cry34Ab1/Cry35Ab1 proteins represent an important seed trait technology having been developed as insect resistance traits in commercialized corn hybrids for control of WCR. The structures of Cry34Ab1 and Cry35Ab1 have been elucidated to 2.15 Å and 1.80 Å resolution, respectively. The solution structures of the toxins were further studied by small angle X-ray scattering and native electrospray ion mobility mass spectrometry. We present here the first published structure from the aegerolysin protein domain family and the structural comparisons of Cry34Ab1 and Cry35Ab1 with other pore forming toxins.

## Introduction

*Bacillus thuringiensis* strains are well-known for the production of insecticidal toxins on sporulation and these proteins are deposited in parasporal crystalline inclusions, closely associated with the spore. To date, many crystal toxins (Cry) have been discovered and these are currently divided into 73 major classes (see http://www.lifesci.susx.ac.uk/home/Neil_Crickmore/Bt/for an updated list). The great majority of these toxins belong to a single structural class of proteins, exhibiting 3 domains in the mature toxin sequence. However, an increasing number of other Cry toxins are structurally and evolutionarily unrelated to this three-domain family. Unfortunately, little is known of their three dimensional structures, limiting our understanding of their mechanisms of action and our ability to engineer the proteins to enhance their function. Amongst the non-three domain Cry toxins, the Cry34Ab1 and Cry35Ab1 proteins from *B. thuringiensis* strain PS149B1 are required to act together to produce toxicity to the western corn rootworm (WCR) *Diabrotica virgifera virgifera* via a pore forming mechanism of action [1], [2], [3]. Very few Cry proteins have been described with activity against WCR [4] and among them the binary mode of action of Cry34Ab1/Cry35Ab1 and related Cry family members is unique. The Cry34Ab1/Cry35Ab1 proteins are important for WCR resistance trait technology, having been introduced to corn hybrids through genetic transformation event DAS-59122-7 to provide protection from WCR feeding in commercialized corn hybrids [5].

Cry34Ab1 is a protein of ∼14 kDa with features of the aegerolysin family (Pfam06355) of proteins that have known ability to interact with membranes to form pores [6] while Cry35Ab1 appears to contain β-trefoil sequences reminiscent of the carbohydrate-binding domain of ricin B subunit (Pfam00652) and is a member of the toxin_10 family (Pfam05431) that includes the binary toxin BinA/BinB, the Cry49Aa1 component of a second binary toxin (Cry48Aa1/Cry49Aa1) from *Lysinibacillus sphaericus*, the Cry36Aa1 protein of *B. thuringiensis* and a hypothetical protein from *Chlorobium phaeobacteroides* [7], [8], [9]. In this study we have elucidated the structures of both Cry34Ab1 and Cry35Ab1 and further probed the consistency of the crystal structure data with their structures in solution using small angle X-ray scattering (SAXS) and native electrospray ion mobility mass spectrometry. We present here the first published structure from the aegerolysin protein family and compare the Cry34Ab1 and Cry35Ab1 protein structures with other pore forming toxins.

## Materials and Methods

### Expression and purification of full length Cry34Ab1 and Cry35Ab1

Full length Cry35Ab1 and Cry34Ab1 toxins were over-expressed in the inclusion body fraction of recombinant *Pseudomonas fluorescens* (*Pf*) [10] and were purified as follows. Whole broth, including *Pf* cells, was frozen at −20°C. To isolate and wash the inclusion bodies, the broth was thawed at 37°C and 200 mL lysis buffer (50 mM Tris-HCl, pH 7.5, 0.2 M NaCl, 5% glycerol, 1 mM DTT 20 mM EDTA and 0.5% Triton X-100) was added for every 60 grams of frozen broth, mixed and centrifuged at 10,000 g for 20 minutes at 4°C. The cell pellet was resuspended at 200 mg cell pellet/mL cold lysis buffer and the cells disrupted by micro-fluidization using a pressure difference of 16,000 psi. Lysozyme was then added to 0.6 mg/mL and the mixture briefly incubated at 37°C, then placed on ice for one hour with stirring. Cell lysis was confirmed by microscopy. Magnesium sulfate was added to 60 mM and DNase I added to 0.25 mg/mL and the mixture incubated overnight at 4°C with stirring. The mixture was gently homogenized using a hand held homogenizer to shear any undigested genomic DNA and centrifuged at 10,000 g for 20 minutes at 4°C. The pellet was washed in lysis buffer and centrifuged three more times.

### Purification and crystallization of Cry35Ab1

Freshly prepared Cry35Ab1 inclusions (100 mg) were solubilized in 100 mL of 50 mM sodium citrate, pH 3.5, precipitated by the addition of 80% ammonium sulfate then isolated by centrifugation at 15,000 g for 15 minutes at 4°C. The pellet was resuspended in 4.0 mL of 15 mM sodium citrate, pH 3.5 and dialyzed against 6 L of the same buffer overnight using a 10,000 MWCO dialysis membrane. The pellet was completely dissolved. This method reliably yielded 60 mg of highly pure Cry35Ab1. The final concentration of Cry35Ab1 used in crystallization experiments was 10–15 mg/mL in 15 mM sodium citrate, pH 3.5. The results from SDS-PAGE analysis and dynamic light scattering scan indicated Cry35Ab1 had reached>90% purity and was monodisperse in solution.

Microbatch crystallization experiments using the Hampton Index screen were set up with the Cry35Ab1 protein. Small crystals appeared within several conditions after two days. After optimization, the best crystals were produced in 0.72 M NaH_2_PO_4_, pH 4.5, 80 mM K_2_HPO_4_ and 0.2 M NaCl with ∼15 mg/mL Cry35Ab1 protein at 16°C.

### Purification and crystallization of Cry34Ab1

In order to achieve a highly concentrated protein solution for crystallization, washed Cry34Ab1 inclusions were dissolved in 7 M urea then refolded using a specially designed dialyzer. This dialyzer has an open-end tube with a 10 K cutoff membrane at the bottom end, which was hung in the center of a micro-centrifuge tube. The membrane allows the solution outside of the dialysis tube to equilibrate slowly into the tube. The small membrane surface limits the rate of diffusion and may also create gradients of components of dialysate in the tube.

About 8.9 mg of Cry34Ab1 powder was initially dissolved in 0.5 mL of 7 M urea, 20 mM potassium phosphate buffer pH 7.8. A 40 µL sample of this protein solution was transferred into the microdialysis apparatus described above and dialyzed against a 3.0 mL solution of 25% (v/v) PEG 400 and 50 mM sodium acetate, pH 4.4, at 16°C. The results from SDS-PAGE analysis and dynamic light scattering scan indicated Cry34Ab1 had reached>90% purity and were monodisperse in solution. After two days, small hexagonally-shaped crystals (0.15×0.15×0.03 mm^3^) were observed on the membrane.

### Data collection and phasing of Cry34Ab1

Data collection on both Cry34Ab1 (Table 1) and full length Cry35Ab1 (Table 2) crystals were carried out at −180°C on a home X-ray system (Rigaku MicroMax-007 X-ray generator and R-AXIS IV^++^ detector). Two different wavelengths of X-ray radiation (Cu Kα, 1.54 Å and Cr Kα, 2.29 Å) were used to collect data sets for structure refinement and enhanced anomalous signal to phase the protein diffraction data. All data sets were processed using the d*TREK data processing package [11].

**Table:** **Table 1.** Cry34Ab1 (4JOX) data processing, model and refinement statistics.

Data processing statistics		
	Native	Pb derivative
Radiation Wavelength	1.54	1.54
Resolution (Å)	22.37–2.15 (2.23–2.15)	26.15–3.00 (3.11–3.00)
Space group	I422	I422
Unit cell length (Å)		
a	100.6	100.3
b	100.6	100.3
c	56.2	56.3
Number of reflections (>1σ)	299,280 (29284)	84,864 (8538)
Unique reflections	14,955 (1500)	5,511 (559)
Average *I*/s (*I*)	17.7(3.5)	17.5 (7.0)
R_merge_ a (%)	9.1 (29.1)	10.0 (31.7)
Completeness (%)	99.8 (99.6)	100.0 (100.0)
FOM after MIR	0.45	
**Model and refinement statistics**		
Resolution range (Å)	12.0–2.15	
Reflections (total)	7271	
Reflections (test)	729	
R_cryst_ b (%)	22.4	
R_free_ b (%)	27.6	
RMSD Bond length (Å)	0.01	
RMSD Bond Angle (°)	1.70	
RMSD Dihedrals (°)	0.10	
Number of protein atoms	915	
Number of water atoms	67	
<B> All protein atoms (Å^2^)	26.7	
<B> Side chain atoms (Å^2^)	27.9	
<B> Main chain atoms (Å^2^)	25.6	
<B> Water molecules (Å^2^)	40.2	
Ramachandran plot^c^	92.1/7.9	

a R_merge_  =  100Σ(h)Σ(i)|I(i)-<I>|/Σ(h)Σ(i)I(i) where I(i) is the **i**th intensity measurement of reflection h, and <I> is the average intensity from multiple observations.

b R_cryst_  =  Σ||**F**_obs_|-|**F**_calc_||/Σ|**F**_obs_|. Where **F**_obs_ and **F**_calc_ are the structure factor amplitudes from the data and the model, respectively. R_free_ is R_cryst_ with 10% of the structure factors.

c Number of residues in favored/additionally favored outlier region. Calculated using PROCHECK [14].

**Table:** **Table 2.** Cry35Ab1 (4JP0) data processing, model and refinement statistics.

Data processing statistics				
	Native		Pt derivative	
Radiation Wavelength	1.54	2.29	1.54	2.29
Resolution (Å)	26.37–1.80 (1.88–1.80)	25.37–2.7 (2.78–2.7)	24.51–2.5 (2.57–2.5)	21.98–2.85 (2.91–2.85)
Space group	P2_1_2_1_2_1_	P2_1_2_1_2_1_	P2_1_2_1_2_1_	P2_1_2_1_2_1_
Unit cell length				
a	48.7	48.6	48.5	48.5
b	65.1	64.3	65.0	64.4
c	117.4	117.2	117.2	117.1
Number of reflections (>1σ)	403,522 (39,620)	122,443 (11937)	36,245 (3593)	107,428 (11890)
Unique reflections	35,100 (4,862)	10,791 (1203)	14,538 (1498)	8,968 (9133)
Average *I*/s (*I*)	21.3 (2.5)	15.3 (5.6)	12.5(3.0)	15.9 (6.5)
R_merge_ a (%)	5.6 (29.2)	8.4 (29.6)	7.6 (27.9)	11.3 (32.8)
Completeness (%)	99.3 (95.3)	94.4 68.4)	96.5 (96.5)	99.7 (94.0)

Model and refinement statistics	
Resolution range (Å)	15.0–1.80
Reflections (total)	31,479
Reflections (test)	3,504
R_cryst_ b (%)	18.1
R_free_ b (%)	23.4
RMSD Bond length (Å)	0.01
RMSD Bond Angle (°)	1.53
RMSD Dihedrals (°)	0.113
Number of protein atoms	3053
Number of water atoms	295
<B> All protein atoms (Å^2^)	25.1
<B> Side chain atoms (Å^2^)	26.7
<B> Main chain atoms (Å^2^)	23.5
<B> Water molecules (Å^2^)	33.3
Ramachandran plot^c^	89.2/10.8

a R_merge_  =  100Σ(h)Σ(i)|I(i)-<I>|/Σ(h)Σ(i)I(i) where I(i) is the **i**th intensity measurement of reflection h, and <I> is the average intensity from multiple observations.

b R_cryst_  =  Σ||**F**_obs_|-|**F**_calc_||/Σ|**F**_obs_|. Where **F**_obs_ and **F**_calc_ are the structure factor amplitudes from the data and the model, respectively. R_free_ is R_cryst_ with 10% of the structure factors.

c Number of residues in favored/additionally favored outlier region. Calculated using PROCHECK [14].

As a novel structure, the multiple isomophous replacement (MIR) method was employed to phase Cry34Ab1 diffraction data. After numerous trials, a Pb-derivatized crystal was prepared by soaking a native Cry34Ab1 crystal in a crystallization solution containing 10 mM lead acetylacetonate for 24 hours. The diffraction data were collected at −180°C (Table 1). One major lead site was found on three Harker sections of an isomorphous difference Patterson map. Two more minor lead sites were found in the isomorphous difference Fourier map of F_PH_ – F_P_ using the phases calculated from the major lead site. Three lead sites were initially used in phase calculation up to 3.0 Å resolution with the program MLPHARE in CCP4 [12]. The figure of merit (FOM) of the single isomorphous replacement with anomalous scattering (SIRAS) phase set was 0.41, while an electron density map calculated with these phases showed clear protein-solvent boundaries but with many broken regions. The anomalous differences of the native data with the initial SIRAS phases were used to generate an anomalous difference Fourier map. A peak with a height of ∼5 sigma above the average value was found in this anomalous Fourier map. It was considered as a sulfur site arising from one of the two methionine residues (the other methionine residue at the N-terminus is disordered and not visible in these maps). The sulfur position and the anomalous difference in the native data were used in further phase calculation. The FOM of the MIR phase set was improved to 0.45. Furthermore, since the anomalous differences of the native data were included as a new independent data set in the phase calculation, it greatly enhanced the power to resolve the phase ambiguity of the initial SIRAS phases from the Pb derivative. The electron density map calculated with these new phases was improved and revealed clear and recognizable regions, such as several β-strands and some large side chain electron density. A solvent-flattening procedure was employed to improve the quality of this electron density map by using program DM [12].

### Data collection and phasing of Cry35Ab1

A platinum derivative of Cry35Ab1 was prepared by an overnight soaking of a native crystal in 20 mM platinum diammine dichloride and the crystallization condition. Crystals were cryoprotected by addition of a final concentration of 20% (v/v) of glycerol to the well condition. Diffraction data sets from both native and heavy-atom derivatives were collected at 100 K with home source X-ray equipment. All the data sets were processed using the d*TREK data processing package [11]. The statistics of data collection are listed in Table 2.

### Structure determination and refinement of Cry34Ab1

The electron density map was used to build an initial model with the program O [13]. The value of the Matthews number indicated that Cry34Ab1 crystals have one molecule per asymmetric unit. The chain tracing and sequence match started at the position of Met54 that was recognized in the anomalous difference Fourier map of the native data set and were extended from there in both directions. Initially, 78 amino acids out of 123 residues were fitted into their densities. The model was refined using the program REFMAC5 [12] and improved by rebuilding after recalculation of the electron density map using weighted combinations of model and MIR phases. Some regions missing in the MIR electron density map gradually appeared in the partial model combined electron density maps. The rigid-body, overall B-factor, individual B-factor and TLS refinement procedure were iterated a number of times to refine the model. The final model was refined to 2.15 Å and contains 117 (from Ala3 to Tyr119) out of 123 amino acids and 67 water molecules. R_cryst_ and R_free_ factors for the final model were 22.4% and 27.6%, respectively. Analysis of the model by the program PROCHECK [14] indicated that 92.1% of the residues fell into the most favored regions of a Ramachandran plot while the remaining 7.9% occurred in additionally allowed regions. The refinement statistics and structure analysis are listed in Table 1. Coordinates and reflection files were assigned the PDB accession code, 4JOX.

### Structure determination and refinement of Cry35Ab1

The initial model was built using the program O [13]. One Cry35Ab1 molecule was determined to be in an asymmetric unit based on calculated Matthews number. The chain tracing and sequence match of Cry35Ab1 was started simultaneously at the position of Cys183 and Met185. They were recognized through their unique densities in the anomalous difference Fourier map of Cr Kα derived native data set. The sequence match was further confirmed by the unique motif of electron densities of Met176, Gly177 and Trp178 and anomalous peak of the sulfur of Met176. About 200 amino acids were fitted into their densities in the first round of map fitting. The model was refined using the program REFMAC5 [12] which includes the procedures of idealization, rigid-body, overall B-factor, TLS and individual B-factor. The electron density map was recalculated using weighted combinations of model and MIR phases. During the refinement process, the electron density was improved in each new map, especially in some uninterpretable regions. The final model contains 378 (from Leu2 to His381) out of 385 amino acids and 295 water molecules. Pro163 and Thr164 were excluded from final model. Some of their electron density was observed in the maps of later cycles, but these two residues cannot be refined into a conformation with both good geometry and density coverage. It may result from their structural location at a loop region with high thermomobility. The final model was refined to 1.80 Å. R_cryst_ and R_free_ factors for the final model were 18.1% and 23.4%, respectively. The analysis of the model by the program PROCHECK [14] indicated 89.2% of the residues fell into the most favored regions of a Ramachandran plot while the remaining 10.8% occurred in additionally allowed regions. The refinement statistics and structure analysis are listed in Table 2. Coordinates and reflection files were assigned the PDB accession code, 4JP0.

### Expression and purification of soluble, truncated Cry35Ab1

A transgenic corn line encoding full length versions of both Cry34Ab1and Cry35Ab1 was jointly developed by Dow AgroSciences and Pioneer Hi-Bred International [15], [16] and sold under the brand name HERCULEX RW. Full length Cry35Ab1 is 44 kDa; however, during characterization of the proteins expressed in transgenic corn, a 40 kDa C-terminal truncation of Cry35Ab1 (trCry35Ab1) was isolated. Interestingly, this 40 kDa form retains both the insecticidal activity and immunoreactivity of the full length Cry35Ab1 [17]. In this study we wished to use this construct to examine the solution state of the truncated molecule in comparison to the crystal structure. In addition, trCry35Ab1 is highly soluble and stable over the time course of experimentation. A plasmid encoding residues 1–354 of Cry35Ab1 (trCry35Ab1; lacking 31 residues at the C-terminus) was transformed into a Dow AgroSciences *P. fluorescens* expression strain [10]. Seed cultures were grown overnight. Production cultures were inoculated with 2% volume of the overnight culture and grown in production media with trace elements and fermented in 2 L controlled bioreactors. Twenty-four hours post inoculation, the cultures were induced with 0.3 mM IPTG. The cells were harvested at 48 hours post-induction by centrifugation. The pellets were stored at −80°C until purification. Routine expression levels are ∼30 grams of soluble trCry35Ab1 per liter of cell culture.

The trCry35Ab1 is expressed in the soluble fraction of the cell lysate. Frozen cell pellets were resuspended in 0.1 M Na acetate, pH 3.3, 1 mM EDTA and 1 mM TCEP (tris(2-carboxyethyl)phosphine). The suspension was sonicated for 30 seconds, followed by a 1 minute rest on ice, three times. After lysis, the lysate was centrifuged at 19,000 rpm for 20 minutes at 4 °C. The supernatant was filtered through a 0.22 µm filter. Purified protein was obtained by using cation exchange chromatography with a Source 30S 16/20 column pre-equilibrated in 0.1 M sodium acetate pH 3.3, and gradient elution with 0.1 M sodium acetate pH 3.3 and 1 M NaCl. The fractions containing trCry35Ab1 were concentrated with 10,000 MWCO 15 mL, Amicon concentrators, centrifuged at 5000 g for 10 minutes. Final samples were filtered through a 0.22 µm filter and applied to a Superdex 75 26/90 column pre-equilibrated in 20 mM sodium citrate, pH 3.3.

### Small angle X-ray scattering

Full length Cry34Ab1 and trCry35Ab1 samples, prepared as described above, were diluted to various concentrations between 2.07 to 6.86 mg/mL in 20 mM sodium citrate pH 3.3. Immediately prior to the data collection, both samples were centrifuged at 14,000 rpm in a tabletop centrifuge for one hour, then filtered through a 0.22 µm syringe filter. Synchrotron scattering data were collected and processed at beamline 5-ID-D at the Advanced Photon Source at Argonne National Laboratories, Illinois, USA.

Data were analyzed using the ATSAS package [18]. Buffer scattering intensities were subtracted from the sample image to remove background scattering using PRIMUS [19]. For the SAXS data, the radius of gyration and the particle distance distribution function, p(r) were evaluated with the GNOM program [20]. Particle shapes were generated using the *ab-initio* software program DAMMIN [21]. Multiple DAMMIN runs were performed (∼25) to check the ‘uniqueness’ of the solution and to generate 25 similar shapes that were combined and filtered to produce an averaged model using the DAMAVER and DAMFILT programs [22].

Cry34Ab1 and Cry35Ab1 crystal structures were docked with the SAXS calculated envelopes using the Chimera program [23]. The C-terminal residues 355–381 of the full length Cry35Ab1 crystal structure were removed for SAXS docking purposes.

### Native electrospray ion mobility mass spectrometry

The behavior of Cry34Ab1 and trCry35Ab1 in solution were probed using native electrospray ion mobility mass spectrometry. Stock solutions of Cry34Ab1 (3.5 mg/mL stored in 20 mM sodium citrate buffer, pH 3.5) and a trCry35Ab1 (3.2 mg/mL stored in 20 mM sodium citrate buffer, pH 3.5) were used for direct infusion under non-denaturing nano-electrospray conditions.

In the case of the Cry34Ab1 sample, the stock solution was diluted 4 – fold with 0.1% formic acid and buffer exchanged into 0.1% formic acid (pH 3.0) using a Zeba spin desalting column (ThermoFisher Scientific) pre-equilibrated with 0.1% formic acid. The buffer exchanged sample was subsequently diluted 5 – fold to give a stock solution of approximately 12.9 µM.

The trCry35Ab1 sample was buffer exchanged without initial dilution into 0.1% formic acid using a Zeba spin desalting column pre-equilibrated with 0.1% formic acid. The resulting buffer exchanged material was diluted 5 – fold to give a stock solution of approximately 15.9 µM.

Electrospray mass spectrometry of the Cry34Ab1 and trCry35Ab1 stock solutions were carried out by directly infusing the proteins with a syringe pump at 500 nL/min with an unheated nanospray inlet. Detection and ion mobility measurements of the proteins was carried out using a prototype ion mobility quadrupole time-of-flight (model 6560 IM-QTOF) mass spectrometer at Agilent Technologies (Santa Clara, CA). This instrument utilizes a drift tube configuration with nitrogen collision gas for ion mobility measurements. The drift tube was operated at 27°C, with 4 Torr of nitrogen collision gas.

For calculation of the measured collisional cross sectional areas (CCS) of the analyzed proteins, the drift tube was calibrated according to the manufacturer's directions using infusion of a colchicine standard (400 m/z, literature value for CCS  = 196.2 Å), and the calibration was confirmed using infusion of a standard of ondansetrone (m/z 294, measured CCS value  = 172.5 Å, literature value  = 172.7 Å) [24]. Measured CCS values for Cry34Ab1 and trCry35Ab1 were calculated using these calibration values with software provided by Agilent.

### Determining collision cross sectional areas by MOBCAL

Theoretical collision cross sectional areas (CCS) of Cry34Ab1 and trCry35Ab1 were calculated using the open source software program MOBCAL [25], [26]. MOBCAL source code was downloaded from the website of Professor M.F. Jarrold's group at Indiana University (http://www.indiana.edu/~nano/software.html) and compiled with Fortran 95 in an in-house Linux work station. The MOBCAL program was further modified to process protein systems up to 15,000 atoms. PDB files of Cry34Ab1 and Cry35Ab1 were used as input files. The calculations were carried out with a uniform charge distribution. A scaling factor of 1.0 was applied throughout the calculations. MOBCAL implements three different types of calculations to derive the CCS area between a protein and helium buffer gas: the projection approximation (PA), the exact hard sphere scattering (EHSS) and the trajectory method (TM). In this study, the PA values are consistently in better agreement with experimental IM-MS measurements. All calculated CCS values from three the methods were included in Table 3.

**Table:** **Table 3.** Measured and theoretical values for CCS for Cry34Ab1 and trCry35Ab1.

					Theoretical		
Protein	component	charge (z)	DT (ms, obs.)	CCS Ω (Å^2^)	PA (Å^2^)	EHHS (Å^2^)	TM (Å^2^)
Cry34Ab1	1	7	27.57	1404.7	1368.6	1720.2	1706.6
	2	7	29.86	1477.5			
	3	8	27.39	1633.9			
	4	9	25.06	1675.9			
	5	9	27.22	1819.1			
	6	9	31.04	2092.9			
	7	9	36.6	2497.0			
	8	10	25.32	1841.1			
	9	10	27.34	2047.1			
	10	10	32.6	2415.3			
	11	10	34.84	2597.5			
	12	10	36.79	2771.9			
	13	11	23.91	1922.0			
	14	11	26.55	2253.2			
	15	11	30.35	2406.4			
	16	11	32.52	2837.2			
	17	11	34.7	2872.5			
trCry35Ab1	1	14	32.66	3466.4	3201.0	4146.0	4211.5
	2	14	34.34	3487.8			
	3	15	32.47	3673.3			
	4	15	32.56	3717.9			
	5	16	31.09	3703.6			
	6	17	30.03	3762.0			
	7	17	36.76	4808.8			
	8	18	29.14	3819.5			
	9	18	35.63	5143.1			

Theoretical values were calculated using the projection approximation (PA), exact hard sphere scattering (EHSS) and trajectory method (TM) with helium as the collision gas. Experimentally measured CCS used nitrogen as the collision gas.

### Protein structure alignment by combinatorial extension

Structures of Cry34Ab1 and Cry35Ab1 were aligned against all the 3D structures in the Protein Data Bank (http://www.rcsb.org/pdb/home/home.do). These alignments were performed using the Combinatorial Extension (CE) algorithm [27]. This method is a fast and accurate way to perform structural alignment against large protein databases. It identifies the optimal alignment between any two structures by defining an alignment path between aligned fragment pairs in the two structures. Similarity between fragment pairs is calculated on the basis of inter-residue distances between the fragments after the superposition. Other structural features like secondary structure, solvent exposure, dihedral angles, etc. are also included to increase the accuracy of the alignment between the fragment pairs. The algorithm provides the sequence identity, r.m.s. of superposition and a Z-score for each alignment.

### Modeling of related proteins

Using the coordinates of Cry35Ab1 and BinB as a template for Cry49Aa1 and Cry34Ab1 as a template for Pam, the possible structures of the related proteins were modeled using Modeller 9.11 [28], [29]. Briefly, for Cry49Aa1 modelling, structure-based sequence alignments were performed using the amino acid sequences of Cry49Aa1, BinA and BinB as template sequences along with the structure of Cry35Ab1 (PDB ID 4JPO) and BinB (PDB ID 3WA1), followed by automated model building and minimization. Manual inspection of clashes and rebuilding of surface loops was performed using Chimera [23]. Final model selection was based on the GA341 score of Modeller [30] and Ramachandran plots.

## Results and Discussion

### Cry34Ab1 and Cry35Ab1 crystal structures

**Figure 1:**
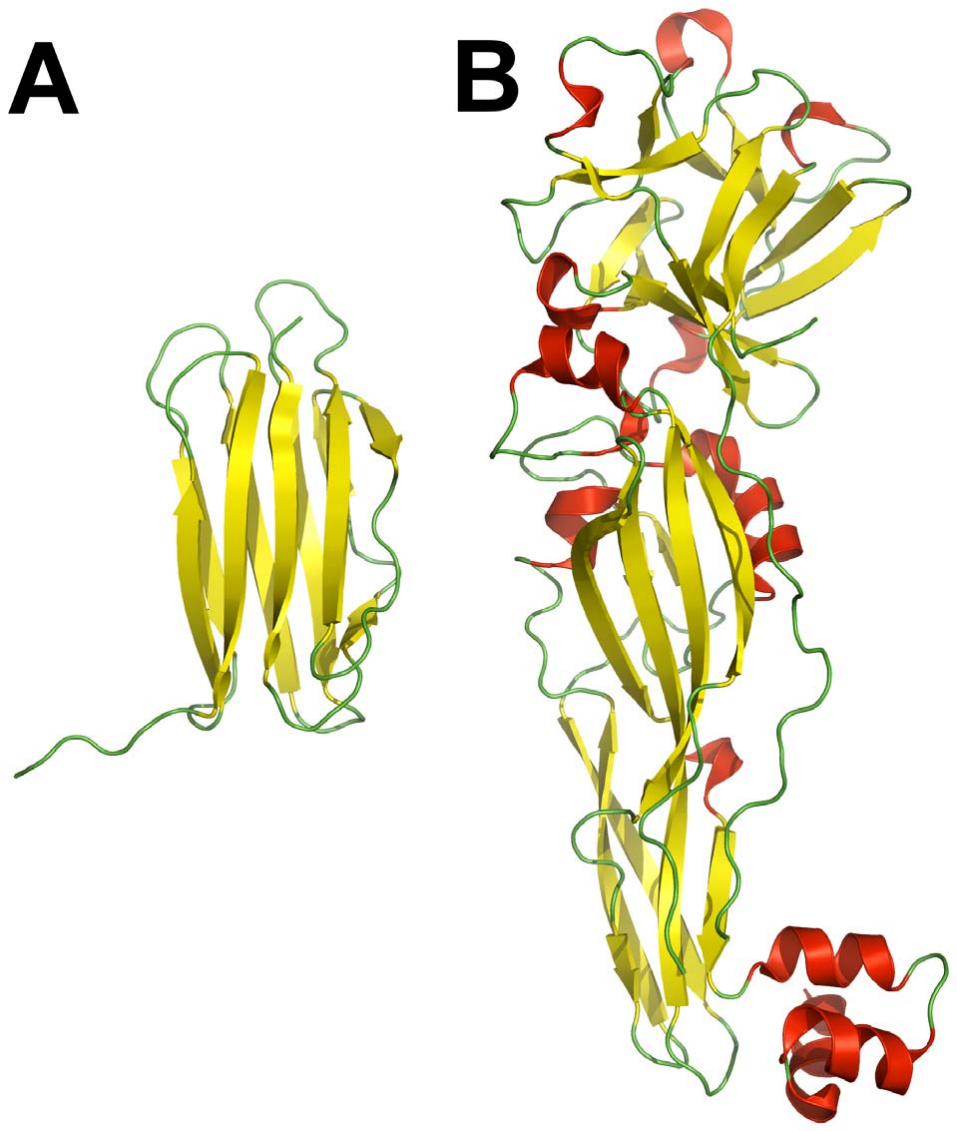
Crystal structures of Cry34Ab1 and Cry35Ab1. (A) The structure of Cry34Ab1 is a β-sandwich of 10 strands. (B) Cry35Ab1 contains two domains. The N-terminal trefoil domain contains α-helices and three β-sheets. The C-terminal domain is terminated with a three helix fold which is not required for activity [17]. This figure, and all subsequent structure representations, were made with PyMOL [66].

The crystal structure of the *Bacillus thuringiensis* Cry34Ab1 protein was refined to 2.15 Å resolution (Table 1). The Cry34Ab1 structure has one distinct structural domain containing 117 amino acids (Figure 1A). The protein folds in a typical β-sandwich conformation, which has two β-sheets packed against each other. β-sheet I containing the N- and C-termini is composed of four β-strands while β-sheet II has five β-strands. All β-strands, except the adjacent N- and C-terminal strands, are antiparallel. N- and C-terminal strands are located at the center of sheet I and parallel to each other. The peptide fragment comprising residues Thr115 to Tyr119 extends beyond its β-sheet toward a symmetry-related molecule within the crystal lattice. The entire β-sandwich has a relatively flat layer-like conformation and two slightly twisted β-sheets. When the side chains are excluded, the distance between the two β-sheets is between ∼7–10 Å. The molecule is ∼45 Å in length and ∼20 Å in width.

As expected, the Cry34Ab1 structure has a very hydrophobic core between the two β-sheets including Val6, Ile8, Val10, Leu18, Trp31, Ile61, Tyr63, Ile71, Leu73, Phe75, Ile96, Val108, Tyr110 and Ile112. The phenol groups of Tyr63 and Tyr110 hydrogen bond with the side chain of Ser106 and the carbonyl oxygen of Thr36, respectively. Residue Trp31 is located at the loop region between β-strand 2 and 3 and its indole group is inserted directly into the core.

**Figure 2:**
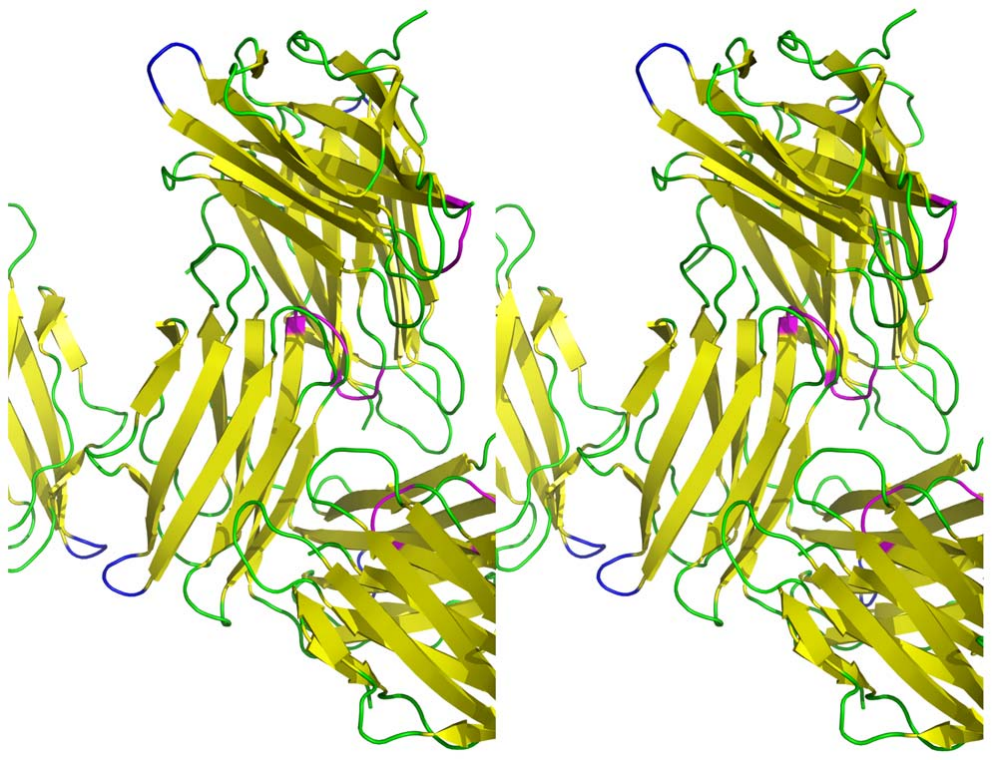
Stereogram of Cry34Ab1 crystal packing. Symmetry related molecules of Cry34Ab1 are shown with loop regions Asn66 to Gln68 colored blue and Gly103 to Gln105 colored magenta. Neither loop region is stabilized by crystal contacts or intramolecular interactions which results in elevated temperature factors and diminished electron density quality.

Nearly every residue in the final model of the Cry34Ab1 structure has well-defined electron density except for residues in two short loop regions (Asn66 to Gln68 and Gly103 to Gln105), which have relatively poorly defined electron density. Both these regions have higher average temperature factors for their main chain and side chain atoms than that of residues calculated over the entire structure. This indicates these loops might have multiple conformations in the crystal and reside in flexible regions due to the lack of crystal contacts to stabilize them (Figure 2).

A total of 67 water molecules were included in the final model of Cry34Ab1. Among these water molecules, ∼60% of them have high temperature factors (>40Å^2^). indicating these positions were partially occupied or otherwise disordered in the entire crystal. One water molecule (HOH1) is located at a special position of a two-fold crystallography axis and participates in hydrogen bonds with the side chains of two symmetry-related His107 residues.

The crystal structure of the Cry35Ab1 protein was refined to 1.80 Å resolution (Table 2). The Cry35Ab1 structure has an elongated rectangular shape with dimensions 42×45×105 Å and is composed of two distinct domains (Figure 1B). Residues Pro163 and Thr164, had no interpretable electron density. The N-terminal domain is a β-trefoil fold, which contains a very hydrophobic core including Ala19, Val30, Leu32, Trp47, Ile59 Trp70, Val72, Ile77, Val79, Trp91, Ile93, Leu123, Trp135, Tyr100, Ile102, Leu110, Ile121, and Leu137.

The next domain contains six helices and three antiparallel β-sheets. A four antiparallel strand β-sheet sits below the N-terminal domain and another two β-strands form a β-sandwich. Within this fold, a π-π stacking interaction is formed between the phenol rings of Tyr231 and Tyr341. Additionally, Tyr341 is also hydrogen-bonded with Tyr229. The hydrophobic side chains of Val219, Leu221 and Met307 cluster around Ile299.

The N-terminal domain and C-terminal domain pack tightly against each other with more than 400 Å^2^ area buried at the interface. The buried region includes hydrophobic residues Ile184, Ile197, Phe50, Phe48, Pro182, Met182, Ile58, Ile52, Ile271. In addition, a hydrogen bonding network exists between the side chains of residues Tyr82 and Glu270, the side chains and main chains of Tyr202 and Thr4 and Asp53 and Thr273, respectively, and the main chains of residues Gly270 and Asp53. These interactions appear to keep domain packing very strong and the conformation of the entire molecule very rigid. The two cysteine residues (Cys67 and Cys187) are present in the interface but their sulfur atoms are 6.1 Å apart, which is too distant to form a disulfide bond. Cys187 is conserved in all the toxins within this family, except Cry36. It is interesting to note that replacement of the Cys187-equivalent residue in BinA (Cys195) drastically reduces its activity [31] while substitution of the equivalent in BinB (Cys241) has no effect [32].

The Cry35Ab1 structure is ended with a C-terminal cluster of three α-helices. The first two helices form a typical helix-loop-helix. The third helix is perpendicular to this helix-loop-helix and the group is held together through a hydrophobic core, consisting of Leu378, Leu353, Leu356, Ala352, Leu375, Val364. Leu353 and Leu356 are the first and last residues of a distinct sequence pattern of four tandem leucines (353-LLLL-356). Due to these structural characteristics, this C-terminal domain is very stable and tightly packed.

### Cry34Ab1 and Cry35Ab1 solution structures

**Figure 3:**
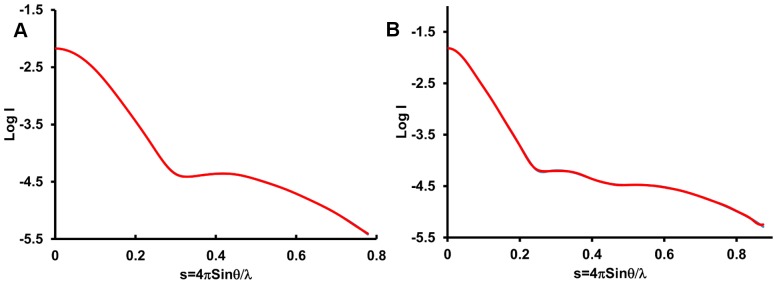
Scattering curve of the SAXS data and experimental fit. (A) Scattering curves for Cry34Ab1 (blue line) from the SAXS experiment and the fit made by the GNOM program [20] (red line) to scattering curve. (B) Scattering curves for trCry35Ab1. The X axis is s in arbitrary units where s = 4πsinθ/λ and the Y axis is the log of intensity in arbitrary units.

**Figure 4:**
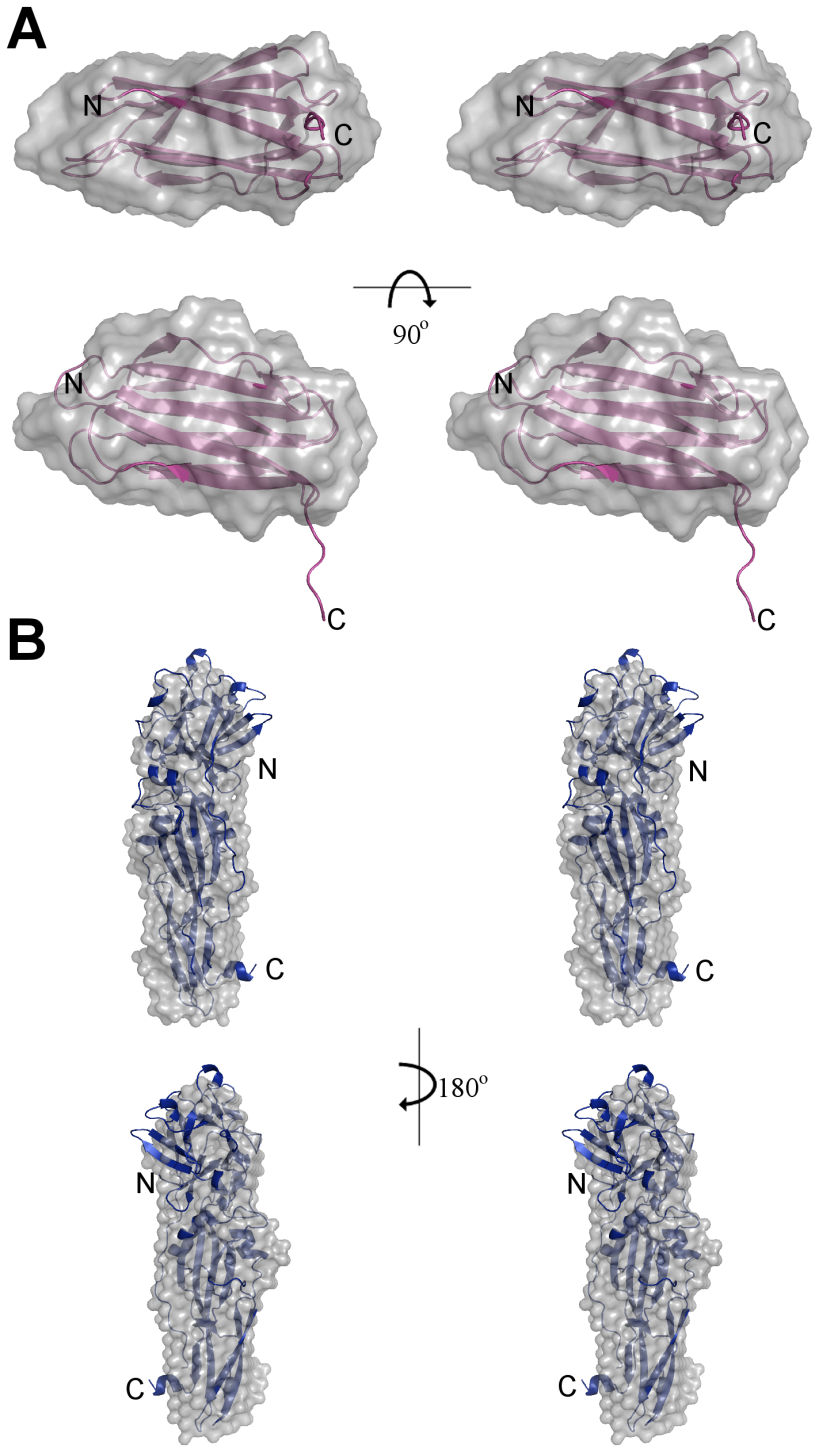
Stereogram of SAXS shapes of Cry34Ab1 and trCry35Ab1. (A) The SAXS calculated envelope of Cry34 matches closely to the crystal structure. The top view is related to the bottom view by 90° rotation to the bottom of the page. (B) The trCry35Ab1 SAXS structures are in good agreement with the Cry35Ab1 crystal structure. No higher order structures or oligomeric states were evident in either the Cry34Ab1 or trCry35Ab1 SAXS data. It is clear that both toxins are monomeric in solution in the absence of a receptor or binding partner.

Analysis of the individual crystal structures suggested that both toxins are monomeric in solution, with no obvious higher order associations based upon content of the asymmetric unit or symmetry related molecules. To confirm the monomeric state in solution and under native conditions, we calculated the solution structures of both Cry34Ab1 and trCry35Ab1 by small angle X-ray scattering. The molecular envelope was generated using the ATSAS software package [18] and superimposed with the crystal structures (Dataset S1 and Dataset S2). The SAXS data indicate that both proteins exist as monomers in these conditions with the predicted radii of gyration of 14.6 and 26.7 Å calculated from the crystal structures of Cry34Ab1 and Cry35Ab1 (with the C-terminal three helix domain removed, consistent with the trCry35Ab1 sequence) respectively, matching closely with those of the SAXS models (14.9 and 25.9 Å, respectively). The overlap of the SAXS envelope and crystal structures (Figure 3) correlates well for both Cry34Ab1 (Figure 4A) and trCry35Ab1 (Figure 4B). It also suggests that the structures remain stable over a range of pH values since this match is seen despite differences in the pH of the crystallization and SAXS conditions (SAXS carried out at pH 3.3 compared to Cry35Ab1 crystallization at pH 4.5 and Cry34Ab1 crystallization at pH 7.8). Removal of the C-terminal 31 amino acids of Cry35Ab1 in the trCry35Ab1 SAXS structure does not appear to alter the core structure of the toxin in solution. When taken together, these finding support a monomeric solution state.

To expand upon the calculated SAXS solution structures, Cry34Ab1 and trCry35Ab1 were further assessed by native electrospray ion mobility mass spectrometry. Experimentally measured values for the collisional cross sectional (CCS) area of both Cry34Ab1 and trCry35Ab1 were produced using nano electrospray of the proteins in 100% water at pH 3.0 (Dataset S3).

Proteins that are ionized under denaturing electrospray conditions typically exhibit a large number of high charge states as a result of the denaturation exposing multiple protonation sites. Ionization under non-denaturing conditions exposes a much smaller number of protonation sites on the surface of the folded protein, resulting in a narrow distribution of conformers with low charge states at high m/z values. Electrospray of both Cry34Ab1 and trCry35Ab1 under these conditions showed a narrow distribution of charge states at relatively high m/z, indicative of the low charge states typically observed for electrospray of intact proteins under non-denaturing (native) conditions [33].

**Figure 5:**
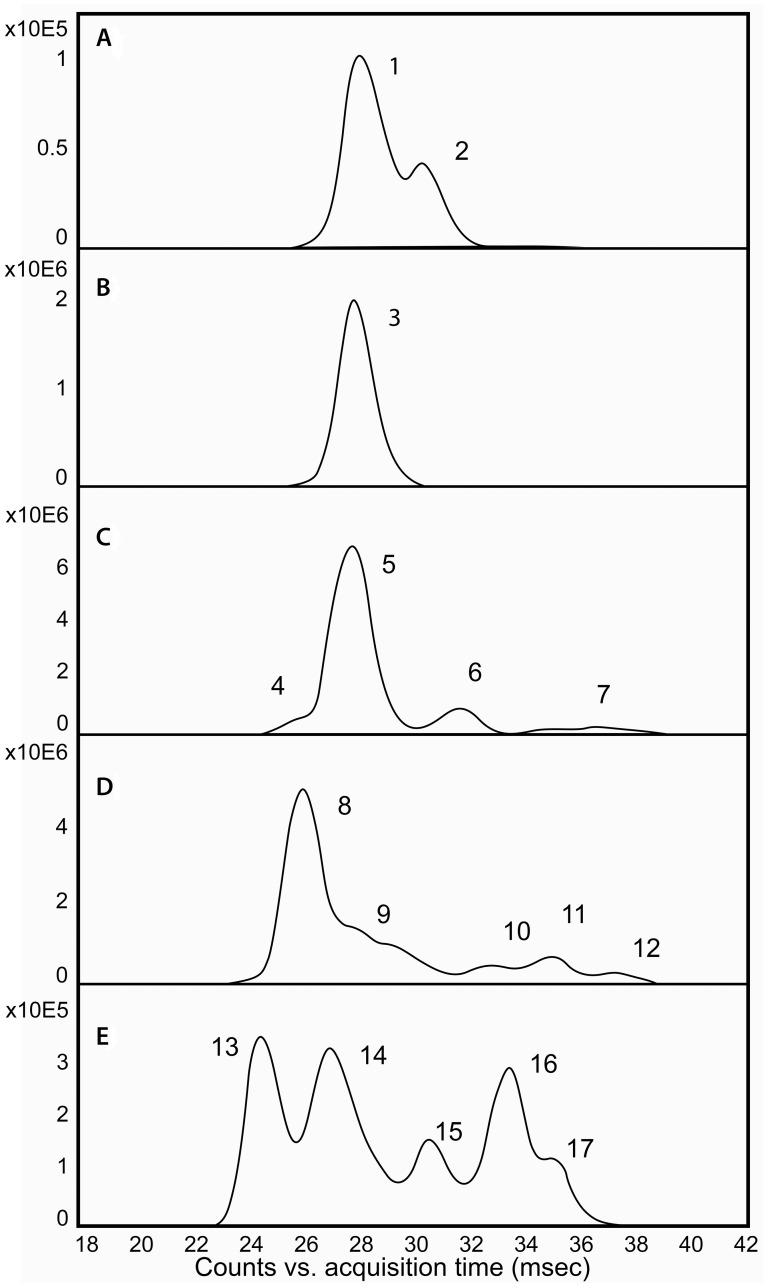
Collision cross sectional profiles of Cry34Ab1. Collision cross sectional profiles of Cry34Ab1. There were five major charge states observed with nanospray. They corresponded to: (A) +7 charge at m/z 1915.62–1941.80; (B) +8 charge at m/z 1680.96–1694.72; (C) +9 charge at m/z 1494.40–1508.25; (D) +10 charge at m/z 1327.13–1387.07; (E) +11 charge at m/z 1221.60–1229.82. The numbered components refer to values reported in Table 3.

The measured drift traces for the five major charge states observed for Cry34Ab1 are shown in Figure 5. The lowest charge state (Figure 5A, z = 7), typically considered to correspond to the most compact conformation of the protein in solution [33], [34], contains two partially resolved populations. The +8 charge state contains a single, uniform CCS. Increasing the charge on the protein from +9 to +11 leads to an increasing number of resolved populations, with the +11 charge state containing at least five partially resolved species. This observation indicates that increasing the charge on the ionized protein results in unfolding of the Cry34Ab1 protein into multiple populations of conformers.

CCS values (Å^2^) in nitrogen gas were calculated [34] from the measured drift values (msec) for each of the major charge states of both Cry34Ab1 and trCry35Ab1 and are reported in Table 3. For reference, the theoretical values calculated using MOBCAL for both Cry34Ab1 and trCry35Ab1 are also reported.

The lowest charge state for Cry34Ab1 has two components with CCS values of 1404 and 1477 Å^2^. Comparison with literature values for experimentally measured CCS using nitrogen gas indicates that these cross sections are similar to what would be expected for a protein of this size [35]. Cry34Ab1 exhibits a very large degree of conformational flexibility as the charge state of the protein increases from +7 to +11, with the CCS increasing more than 2–fold from 1404 Å^2^ to 2872 Å^2^ (Table 3). This increase in cross sectional area as the number of charges on the protein increases in the gas phase is typically considered to arise from coulombic repulsion of the positive charges as solvent is stripped away from the protein [33], [34]. In the context of the crystal structure, the flexibility of Cry34Ab1 observed with the native electrospray ion mobility is consistent with an increased solvent exposed surface area to packed core ratio.

The trCry35Ab1 also shows a narrow distribution of low charge states (+14–+18) at high m/z values (>2000 m/z). As with Cry34Ab1, the lowest charge state exhibits two partially resolved components, with CCS values of 3466 and 3487 Å^2^. Based on calibrated values for protein standards [35], these values are also consistent with what would be expected for a protein of this size. Based on the number of different conformers observed as the charge state increases from +14 to +18 (Table 3), trCry35Ab1 appears to be more stable in the gas phase compared to Cry34Ab1 due to its increased size.

The theoretical values calculated with MOBCAL assume helium as a collision gas [25], [26], and thus are not directly comparable with the experimentally measured values using nitrogen. Of the three methods used to calculate theoretical CCS values using MOBCAL, the TM method is considered to be the most reliable and accurate [34]. In addition, the theoretical value calculated for trCry35Ab1 is derived from the crystal structure data of the full length protein, whereas the experimentally measured value is from the truncated form which is missing 31 residues from the C-terminus of the protein.

In comparing the experimentally measured CCS values for both the smallest conformers of Cry34Ab1 and trCry35Ab1 with the theoretical values from MOBCAL, the experimental values are consistently smaller. This agrees with previous observations [34] and is considered to be a reflection of partial collapse of the protein structure during desolvation.

Based upon the crystallographic, SAXS and mass spectrometry data collected, it is clear that both toxins are monomeric in solution and remarkably stable. However, higher-order oligomeric organization of Cry34Ab1 and Cry35Ab1 in the presence of any putative membrane receptors cannot be ruled out.

### Protein structure alignment by combinatorial extension

Structural alignment of protein structures in combination with sequence homology studies provides additional insight into the evolutionary relationships and modes of action of proteins. To this end, 3D structures of Cry34Ab1 and Cry35Ab1 were aligned against all the 3D structures in the Protein Data Bank (http://www.rcsb.org/pdb/home/home.do). Table 4 lists the top 10 unique structures in the pdb that have a threshold z-score of 4 or higher with the Cry34Ab1 structure. It also includes the CATH description for the protein where available. Table 5 provides a similar analysis for the Cry35Ab1 structure.

**Table:** **Table 4.** Combinatorial extension analysis of PDB submitted structures against Cry34Ab1 coordinates.

PDB ID	Name	RMS superposition	Z-score	% sequence identity	CATH Classification
1o72	Cytolysin Sticholysin II	3.47 Å	5.2	16.10%	Mainly Beta; Sandwich; Mutm (Fpg) Protein
3lim	Fragaceatoxin C	3.33 Å	5	17.70%	N/A
1iaz	Equinatoxin II	4.15 Å	5	15.00%	N/A
2qqp	Providence Virus	4.43 Å	4.6	12.00%	N/A
2qsv	Protein of unknown function	2.88 Å	4.6	10.50%	Mainly Beta; Sandwich; Immunoglobin-like; PapD-like
3a57	Thermostable direct hemolysin	2.81 Å	4.6	8.70%	N/A
1bci	Phospholipase	4.08 Å	4.6	8.10%	Mainly Beta; Sandwich; Immunoglobin-like; C2-domain Calcium/lipid binding domain
2xc8	Gene 22 product of the *Bacillus subtilis* SPP1 Phage	7.64 Å	4.6	7.10%	N/A
3l9b	Otoferlin C2A	4.52 Å	4.6	6.90%	N/A
3i6s	Plant subtilisin-like protease SBT3	2.64 Å	4.4	13.50%	N/A

Results of the top 10 unique hits ranked according to Z-score.

**Table:** **Table 5.** Combinatorial extension analysis of PDB submitted structures against Cry35Ab1 coordinates.

PDB ID	Name	RMS superposition	Z-score	% sequence identity	CATH Classification
3wa1	BinB	2.73 Å	7.1	17.2%	
1ups	β-galactosidase	7.10 Å	6.1	15.10%	Mainly β-sandwich; Trefoil; Jelly Rolls
2f2f	Cytolethal distending toxin	2.45 Å	6.1	12.80%	Mainly β-sandwich; Trefoil; Jelly Rolls; Alpha-Beta; 4-Layer Sandwich; DNase I
1qxm	Hemagglutinin component (HA1)	1.99 Å	6	22.70%	Mainly β-sandwich; Trefoil; Jelly Rolls
3ef2	Mushroom lectin	2.29 Å	6	19.90%	N/A
2y9g	β-trefoil lectin binding domain	2.60 Å	6	13.00%	N/A
2vsa	Mosquitocidal holotoxin	2.28 Å	5.9	19.70%	N/A
3nbd	Ricin-B like lectin domain	3.11 Å	5.9	19.40%	
1dfc	Fascin	2.91 Å	5.9	13.50%	Mainly Beta; Trefoil;
2yug	FRG1	2.50 Å	5.9	11.20%	N/A

Results of the top 10 unique hits ranked according to Z-score.

The Cry34Ab1 β-sandwich architecture is most similar to a superfamily of sea anemone toxins known as actinoporins including equinatoxin [36], [37], sticholysin II [38] and fragaceatoxin C [39]. Actinoporins are small, approximately 20 kDa, cytolytic proteins found in sea anemone venom. Cry34Ab1 is also structurally similar to *Vibrio parahaemolyticus* thermostable direct hemolysin [40].

Cry35Ab1 has an overall structure that is very similar to the recently described structure of BinB [41]. Both structures have an N-terminal domain with two QxW repeats and a second domain consisting of extended antiparallel beta sheets. Structural homology of BinB and proteins containing similar β-trefoil lectin-like domains are identified by high Z-scores in Table 5.

### Cry34Ab1 structure comparisons

**Figure 6:**
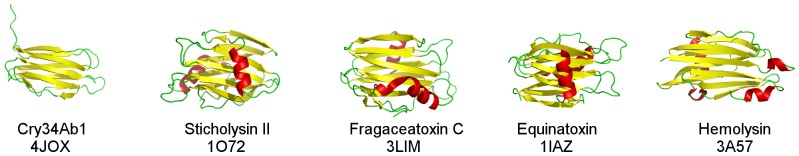
Comparison of proteins structurally related to Cry34Ab1. Cry34Ab1 is structurally related to a wide variety membrane-interacting proteins as assessed by combinatorial extension. All structures compared are comprised of a conserved beta-sheet core and varying loop regions.

The Cry34Ab1 structure is clearly related to other membrane-interacting proteins with a beta sandwich fold, including actinoporins and hemolysin (Figure 6). Both actinoporins and hemolysin form tetrameric pores in lipid membranes [42]. The molecular mechanism of actinoporins has been extensively studied (reviewed in [43]). Actinoporins show strong specificity for sphingomyelin and form pores in membranes containing sphingomyelin. A key feature of actinoporin mechanism of action is the insertion of the N-terminal α-helical segment that precedes oligomerization and pore formation [44]. The actinoporin N-terminal α-helix is necessary for pore formation. Cry34Ab1 does not contain an analogous N-terminal helical structure, implying differences in membrane interaction mode of action when compared to actinporins.

**Figure 7:**
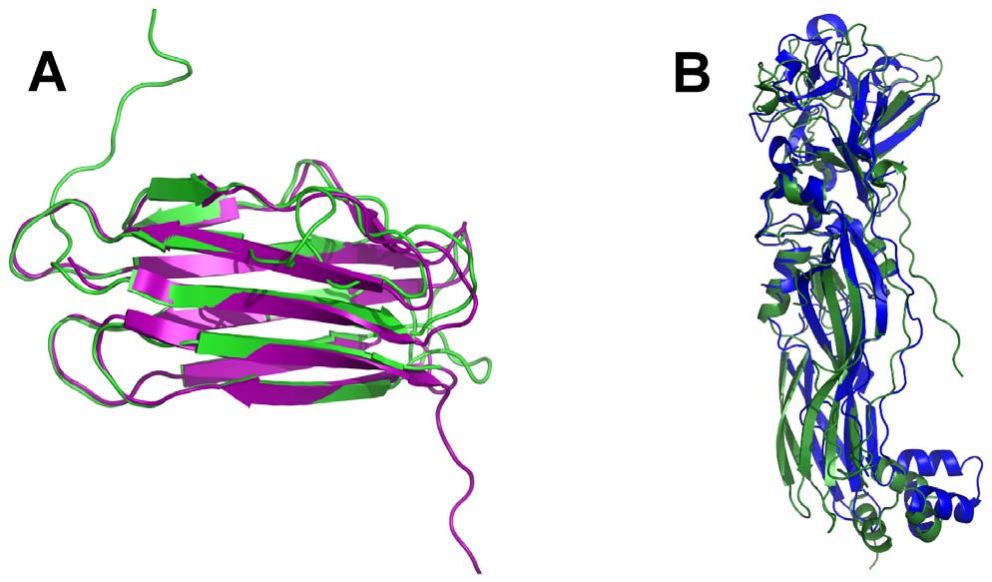
Structural superpositions of Cry34Ab1 and Cry35Ab1 with calculated theoretical models of Pam and Cry49. (A) Overlay of the Cry34Ab1 structure (purple) with a model of Pam (green). (B) Overlay of the Cry35Ab1 crystal structure (blue) with a model of Cry49 (green). See text for details.

Further, Pfam analysis indicates that Cry34Ab1 appears to be a member of the Aegerolysin protein family [6]. The β-sandwich fold exemplified in Cry34Ab1 is common among other cytolytic proteins found in nature including necrosis and ethylene-inducing peptide 1 (Nep1)-like proteins (NLPs) from microbial plant pathogens [45] and fungal fruit lectins [46]. Cry34Ab1 protein also shows some similarity to the Pam protein of *Photorhabdus asymbiotica* [47]. This similarity is sufficient to allow modeling of the Pam sequence based on the Cry34Ab1 template (Figure 7A). The model shows a similar structure but some of the β-strands appear shorter and one strand of the five-strand sheet shows a loop in the Pam model.

### Cry35Ab1 structure comparisons

It is clear from primary sequence alignments that Cry35Ab1 is a member of the toxin_10 family that includes BinA, BinB, Cry36Aa1 and Cry49Aa1. The 3D structure of Cry35Ab1 gives us some insight into the structures of these related proteins as demonstrated by comparison to BinB and the theoretical model of Cry49Aa1. Building this model on both Cry35Aa1 and BinB templates resulted in a model with a probability in excess of 95% as judged by a GA341 score of 0.92133 (where a value>0.7 generally indicates a reliable model, defined as ≥95% probability of a correct fold).

The N-terminal 40 amino acids in the Cry49Aa1 sequence appeared as a flexible region projecting beyond the extended the N-terminus of the BinB structure and could not be modelled reliably and, therefore, was removed from the structure shown in Figure 7B. Cry49Aa1 clearly shows significant similarity to Cry35Aa1, particularly in the core, β-sheet region of the C-terminal domain (Figure 7B). The high proportion of β-sheet structure in Cry35Ab1 is consistent with CD analysis of the related BinA protein that indicated a high proportion of β-sheet and little α-helix [48], [49], [50].

**Figure 8:**
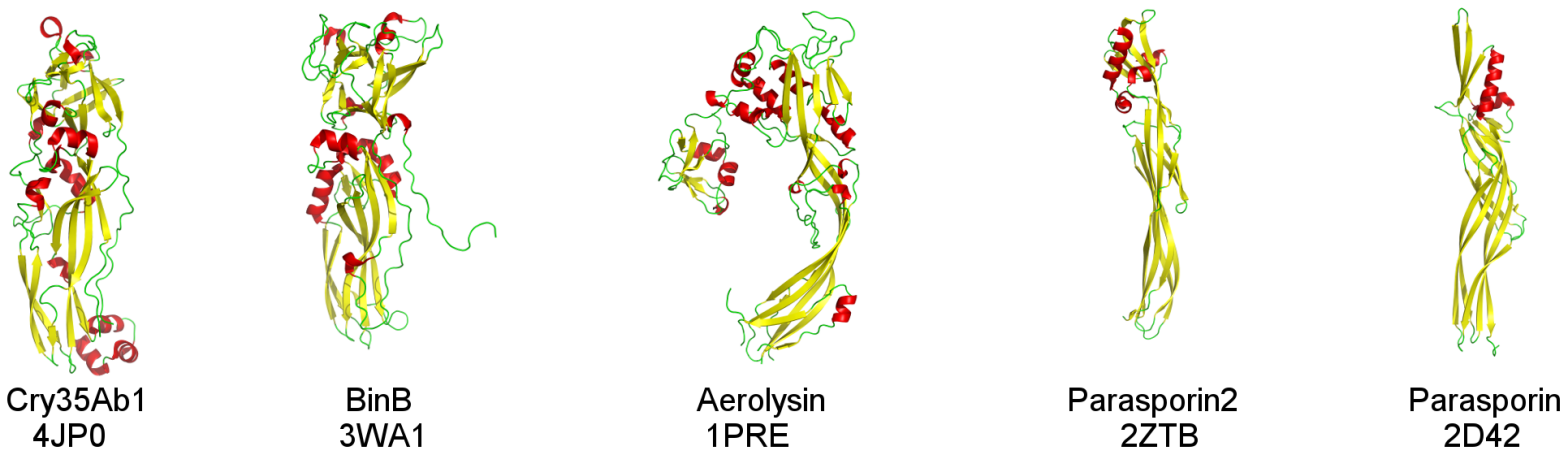
Comparison of proteins structurally related to Cry35Ab1. Cry35Ab1 is structurally related to a wide variety pore-forming proteins as assessed by combinatorial extension. All structures contain a conserved beta-sheet core and varying loop regions.

Cry35Ab1 is also related to a 41.9 kDa toxin_10 family protein found in the genomes of a number of *Bacillus cereus* and *B. thuringiensis* strains although, to date, no toxicity has been found for this protein, which has only been tested thus far on lepidopteran targets [51]. In addition, the structure of Cry35Ab1 shows interesting similarities to several other toxins that show no significant relationship at the primary sequence level yet, like Cry35Ab1, are predominantly composed of β-sheets arranged in extended structures. These include the structure of aerolysin (PDB accession number 1PRE), parasporin 4 (PDB accession 2D42; also known as Cry45Aa1) and parasporin 2 [52], [53] (PDB accession numbers 2D42 and 2ZTB; also known as Cry46Aa1) (Figure 8). Membership of the β-pore forming toxin family that includes aerolysin is consistent with Cry35Aa1 causing toxicity by participating in pore formation [1]. The mechanism of action of this family of toxins involves the oligomerisation of individual subunits followed by structural rearrangements that must occur for the penetrating β-sheet pore structures to enter the membrane. Parasporins, like Cry proteins, are produced as crystalline inclusions by *B. thuringiensis* strains but, to date, have no reported toxicity to invertebrates however, have demonstrated anti-cancer activity [54], [55].

The structures of Cry34Ab1 and Cry35Ab1 also show striking similarity to the structures determined for Cry37Aa1 and Cry23Aa1 respectively [56], despite little primary sequence identity. Cry37Aa1/Cry23Aa1, like Cry34Ab1/Cry35Ab1, represents a two-component toxin active against certain coleopteran targets [57]. Cry23Aa1 appears to be a member of a family of proteins that include *B. thuringiensis* Cry38Aa1, a protein of unknown activity encoded by a gene linked with the *Cry34Aa1* and *Cry35Aa1* operon [58]. Cry33Aa1, Cry45Aa1, Cry15Aa1 and the related proteins, Bti34 and Bti36, are described as having antibacterial activity by Revina *et al.* [59]. In addition to these *B. thuringiensis* proteins, Cry23Aa is also related to the Mtx2, Mtx3 and Mtx4 proteins of *L. sphaericus* [7] and to *Aeromonas hydrophila* aerolysin [56]. Given the structural relationship of Cry23Aa1 to Cry35Aa1 and its related crystalline proteins Cry36Aa1, Cry49Aa1, BinA and BinB, nature seems to have adapted the extended antiparallel β-sheet structure extensively in the production of insecticidal toxins in the *Bacillus* and *Lysinibacillus* genera.

The Cry35Ab1 protein contains two repeats of the QxW motif found within the N-terminal, β-trefoil domain. Such features may be involved in lectin-like binding in toxins such as ricin B subunit [2]. The lectin-like domains of aerolysin and pertussis toxin have been proposed as conserved receptor binding domains [60]. Effects of sugar groups on the toxicity of the *L. sphaericus* Bin toxins have been reported previously [61], [62] although these proteins lack the QxW motif in β-trefoil structures. BinA, BinB and Cry49Aa1 do contain an occurrence of the QxW motif in the β-sheet core of the C-terminal domain as does Cry35Ab1 although this occurrence of the motif is absent from the much more closely-related Cry35Aa1 sequence. In BinA, substitution of Trp222 in this feature, equivalent to Trp211 of Cry35Ab1, results in loss of activity although the protein is still able to permeabilize liposomes [63]. Ricin-B-like lectin repeats were also noted in the 41.9 kDa *B. thuringiensis* protein [51] although there are no QxW motifs in this protein.

The Cry35Ab1 three α-helical C-terminal motif is not required for insecticidal activity [17]. Moreover, removal of this motif to create trCry35Ab1 shifts expression entirely from the insoluble to the soluble fraction of the cell lysate of our heterologous *Pseudomonas fluorescens* expression system. This raises the possibility of a role in the formation of parasporal inclusions by functioning as an inclusion anchor or a protein: protein interaction domain.

### Cry34Ab1 and Cry35Ab1 function

The structural similarities of Cry34Ab1 and Cry35Ab1 to other membrane-binding or pore forming proteins described above suggest several possibilities for a mechanism of action wherein either Cry34Ab1 and/or Cry35Ab1 might possibly initiate pore formation. However, the mechanism of interaction by which Cry34Ab1 and Cry35Ab1 function as a binary toxin is yet unknown. Both proteins have structural features allowing for conformational changes during putative pore formation event. What is currently known regarding mechanism of action is that Cry35Ab1 binds to WCR brush border membrane vesicles and Cry35Ab1 binding is dramatically enhanced by the presence of Cry34Ab1 [64] suggesting formation of a protein complex that results in pore formation. Presence of the N-terminal lectin domain on Cry35Ab1 suggests that binding to membrane glycoproteins might be involved, as has been suggested for BinB [41]. However, because iodo-radiolabelling Cry34Ab1 reduced its biological activity, its role is more difficult to probe and therefore direct interaction of Cry34Ab1 with putative receptors cannot be ruled out. In fact, Cry34Ab1 has biological activity alone, albeit at a much reduced level compared to the binary toxin [65].

In conclusion, the Cry34Ab1 and Cry35Ab1 protein structures presented here, while sharing structural similarity with other pore forming toxins, are novel among proteins developed for corn rootworm resistance traits. This structural information provides the basis for experimentation aimed at dissecting the Cry34Ab1/Cry35Ab1 binary toxin mode of action and for protein engineering aimed at improving insecticidal properties of the proteins.

## Supporting Information

Dataset S1 **Processed Cry34Ab1 SAXS data.**
(DAT)

Dataset S2 **Processed trCry35Ab1 SAXS data.**
(DAT)

Dataset S3 **Processed Cry34Ab1 and trCry35Ab1 native electrospray ion mobility mass spectrometry data sets.**
(XLSX)
